# Technology-Based Fall Risk Assessments for Older Adults in Low-Income Settings: Protocol for a Cross-sectional Study

**DOI:** 10.2196/27381

**Published:** 2021-04-07

**Authors:** Ladda Thiamwong, Jeffrey R Stout, Joon-Hyuk Park, Xin Yan

**Affiliations:** 1 College of Nursing University of Central Florida Orlando, FL United States; 2 Disability, Aging and Technology Cluster University of Central Florida Orlando, FL United States; 3 School of Kinesiology and Physical Therapy College of Health Professions and Sciences University of Central Florida Orlando, FL United States; 4 Mechanical and Aerospace Engineering Department University of Central Florida Orlando, FL United States; 5 Department of Statistics and Data Science University of Central Florida Orlando, FL United States

**Keywords:** body composition, falls, risk assessment, technology, wearable devices, accidental falls, fear

## Abstract

**Background:**

One-third of older adults have maladaptive fall risk appraisal (FRA), a condition in which there is a discrepancy between the level of fear of falling (FOF) and physiological fall risk (balance performance). Older adults who overestimate their physiological fall risk and report a high FOF are less likely to participate in physical activity. Limited data suggest that the association among FOF, body composition, and physical activity intensity differs by fear severity.

**Objective:**

This study aims to examine the associations among FRA, body composition, and physical activity using assistive health technology, including the BTrackS balance system, bioelectrical impedance analysis, and activity monitoring devices. This study also aims to examine the feasibility of recruitment and acceptability of technologies and procedures for use among older adults in low-income settings.

**Methods:**

This cross-sectional study will be conducted in older adults’ homes or apartments in low-income settings in Central Florida, United States. Following consent, participants will be contacted, and our team will visit them twice. The first visit includes questionnaire completion (eg, sociodemographic or FOF) and balance performance test using the BTrackS balance system. The participants will be stratified by the FRA matrix. In addition, they will perform hand grip strength and dynamic balance performance tests. Participants will then be asked to wear the ActiGraph GT9X Link wireless activity monitor on the nondominant wrist for 7 consecutive days. The second visit includes body composition testing and a structured interview about the acceptability of the technologies and procedures.

**Results:**

Ethical approval was obtained from the institutional review board of the University of Central Florida (protocol number 2189; September 10, 2020). As of December 2020, participation enrollment is ongoing and the results are expected to be published in Summer 2022.

**Conclusions:**

Accurate FRA is essential for implementing physical activity programs, especially in older adults with low income. This study will provide data for developing technology-based fall risk assessments to improve participation in physical activity, thus enhancing healthy longevity among older adults in low-income settings.

**International Registered Report Identifier (IRRID):**

PRR1-10.2196/27381

## Introduction

### Background

More than 15 million (30%) older adults in the United States have income below 200% of the poverty line [1,2]. The poverty rate increases with age and is higher among women, Black and Hispanic individuals, and individuals with poor health [1]. Older adults who live in low-income communities are less likely to engage in physical activity (PA) [3-5], defined as any bodily movement produced by skeletal muscles that results in energy expenditure [6]. Lack of PA is related to chronic conditions and reduced quality of life among older adults with low income [7,8]. Limited data suggest that older adults who overestimate their fall risk and report fear of falling (FOF) are less likely to participate in PA, and the association between FOF and PA intensity differs by fear severity [9]. Changes in body composition also have a significant effect on physical functions and quality of life [10]. However, these studies did not include older adults from low-income settings, who were more likely to report falls in the previous year [11].

One-third of older adults have maladaptive fall risk appraisal (FRA), a condition in which there is a discrepancy between the perceived fall risk (levels of FOF) and physiological fall risk (balance performance) [12,13]. Measuring FRA in older adults can be challenging because of self-report bias and cognitive deficit [14,15]. Using both subjective and objective measures provides better fall risk assessment among older adults [15,16]. Thus, we developed *an FRA matrix*, a graphical grid categorizing the levels of FOF and balance performance into 4 quadrants: (1) *rational FRA* (low FOF and normal balance), (2) *incongruent FRA* (low FOF despite poor balance), (3) *irrational FRA* (high FOF despite normal balance), and (4) *congruent FRA* (high FOF and poor balance) [12]. In our pilot study (n=102), we measured FOF using a questionnaire and balance performance using a portable and novel force balance plate (BTrackS Assess Balance System [BBS], Balance Tracking System, Inc). We found that 40% of older adults had maladaptive FRA. In this group, 19% of patients with incongruent FRA and 30% with irrational FRA reported falling in the past year [12]. Older adults with congruent FRA or high fear-high physiological were 2.14 times more likely to fall than those with rational FRA or low fear-low physiological [17]. However, our sample included only 11% of older adults with low income.

### Objectives

Body composition (eg, obesity or low relative skeletal muscle mass) and decline in muscle strength have been associated with FOF, functional impairment, and disability in older American adults [18-20]. Higher BMI and percentage of body fat were associated with poor physical function, whereas the percentage of appendicular lean mass was associated with better physical function (eg, walking or balance test) [19]. Older adults with higher daily PA have better physical function than those who engage in less PA [21]. Although BMI is the most widely used measurement to classify overweight and obesity statuses, it is prone to measurement error and does not consider body fat distribution and skeletal muscle mass [22]. We will use the bioelectrical impedance analysis (BIA) device, which measures body composition and has established normative data among older adults [23].

Research has not examined the associations among body composition, FRA, and PA using assistive health technology (AHT), which applies organizational knowledge, skills, procedures, and systems to improve functioning [24]. This study examines the associations among body composition, FRA, and PA using AHT, including BIA [25], BBS [26,27], and activity monitoring devices [28]. It also aims to examine the feasibility of recruitment and acceptability of technologies and procedures for use among older adults in low-income settings.

## Methods

### Study Design

This is a cross-sectional study.

### Settings

This study will be conducted in older adults’ homes or apartments in low-income settings in Central Florida, United States. Collectively, approximately 300 older adults are available for screening at the planned sites.

### Study Participants

We will enroll a sample of 120 participants, if they meet all of the following inclusion criteria: (1) aged ≥60 years, (2) have a low income (using poverty thresholds for 2019 by family size and the number of children aged <18 years, published by the US Census Bureau) [29], (3) have no marked cognitive impairment (memory impairment screen score ≥5 [30-32]), and (4) live in their own homes or apartments. Exclusion criteria were as follows: (1) a medical condition precluding balance test (eg, inability to stand on the balance plate) or PA (eg, shortness of breath or feeling pressure when performing PA), (2) currently receiving treatment from a rehabilitation facility, or (3) having medical implants (eg, pacemakers).

### Consideration of Sex and Other Relevant Biological Variables

In our pilot study, 66% of the participants were women (mean 77, SD 7.6 years). We found that men tend to cluster in rational and incongruent FRA groups, whereas women were widely distributed among 4 groups (rational, incongruent, irrational, and congruent). We expect that 65% to 70% of our sample will be women. We will explore the differences between men and women and between age groups (<75 years vs >75 years) to assess whether sex and age as biological variables contribute to the findings. 

### Variables and Instruments

The study instruments consist of objective and subjective measures (Table 1).

**Table 1:** Description of study variables and instruments.

Variable and instrument				Description
**Participant’s characteristics**				
	Sociodemographic and medical history	Self-report questionnaire	For example, age, gender, living status, education level, socioeconomic status, perceived general health, comorbidities, mobility problems, medication use, and number of falls in the past year	
	Depressive symptoms	Patient Health Questionnaire-9 [33,34]	A total of 10 items (eg, feeling tired) on a 4-point scale; measures symptoms of depression within the past 2 weeks; total scores range 0-27; and scores ≥10 indicate moderate depression [33]. Cronbach α=.89 among older adults [33,34]	
	Cognition	MIS^a^ [30-32]	The MIS is a widely used test of cognitive function and screening Alzheimer disease among older adults. It is a 4‐minute, 4‐item delayed free and cued recall memory test with controlled learning and high discriminative validity. The maximum score for the MIS is 8, and a score of 5-8 indicates no cognitive impairment and score ≤4 indicates possible cognitive impairment	
FOF^b^		Short FES-I^c^ [35]	A total of 7 items (eg, going in or out of a chair) on a 4-point scale; measures concerns about the possibility of falling when performing 7 activities (eg, getting dressed) [36]. Total scores range 7-28 [35]. Higher total scores=higher FOF [37]. Scores 7-10=low concern about falling; scores 11-28=high concern about falling [36,38]. The short FES-I has been validated in community-dwelling older adults [36]. Cronbach α=.97 and ICC^d^=0.979 among older adults [39]	
Balance performance		BTrackS balance system [26,27]	This test consists of 4, 20-second trials. For each trial, the participants will stand as still as possible on the BTrackS Balance Plate with hands on their hips and eyes closed	
Balance performance		Sit-to-stand test [40,41]	A sit-to-stand test has been widely used to assess balance and predict falls and was reliable and validated in community-dwelling older adults [40,41]	
Hand grip strength		A hand grip dynamometer (JAMAR 5030J1)	Hand grip strength will be measured in kilograms as maximal isometric force achieved on a hand grip dynamometer (ICC=0.959; SEM^e^=3.1 kg)	
Body composition		Bioelectrical impedance analysis: InBodys10 device [25]	Participants will remove socks, shoes, and metal objects before the body composition testing. The touch-type electrodes will be placed to the left and right ankles, middle fingers, and thumbs. Participants will hold the position for 1 minute and then remove the touch-type electrodes	
Physical activity		ActiGraph GT9X Link wireless activity monitors (ActiGraph LLC) [28]	Participants will be asked to wear the activity monitoring device on the nondominant wrist for 7 consecutive days. They will be instructed to remove the monitor for imaging studies	
Acceptability of technology		Evaluation form of acceptability of technology and procedures	Older adults’ evaluation of the devices, procedures, and instruments; recommendations for inclusion of older adults in future research	

^a^MIS: memory impairment screen.

^b^FOF: fear of falling.

^c^FES-I: Fall Efficacy Scale International.

^d^ICC: intraclass correlation coefficient.

^e^SEM: standard error of measurement.

*Balance performance* will be assessed using the BBS [26,27]. The BBS includes a portable BTrackS balance plate (BBP) and BTrackS Assess Balance software running on a computer device. The BBP dimensions are 15.5 × 23.5 × 2.5 in, weight 6.58 kg, and is operated on Windows 7 or higher via a USB port (Food and Drug Administration approved). During the test, a piece of sturdy furniture or a standard walker will be placed within the participant’s reach to reduce the risk of FOF contaminating the performance and to enable even frail people to participate [42]. The software uses the BBS normative database to compare the individual with others in the same age group. The BBS score is dependent on age and sex, but not body size, so that the percentile rankings can be determined across various age groups and for men and women separately [43]. A scale from 0 to 100 represents the percentile ranking of the BBS. A score of 0 to 30 indicates low fall risk (normal balance) and ≥31 indicates moderate-to-high fall risk (poor balance) [43]. The BBS has excellent validity using Pearson correlations (*r*>0.90) and high test-retest reliability (intraclass correlation coefficient [ICC]=0.83) [44].

*Body composition* will be assessed using a direct segmental multifrequency BIA using the InBodys10 device [25]. The BIA is manufactured by Biospace Corporation Limited. The BIA InBody S10 measures impedance at 6 different frequencies (1, 5, 50, 250, 500, and 1000 kHz) for each body segment (right arm, left arm, trunk, right leg, and left leg). The spectrum of electrical frequencies is used in the manufacturer’s equation to estimate fat mass, muscle mass, and body water levels. There are no risks, no dunking, no pinching, and no discomfort associated with the use of BIA. The test duration is 1 to 2 minutes. The reliability of the BIA test-retest was high, with an ICC of 0.89 [45]. 

PA will be measured using activity monitoring devices. All older adults will wear ActiGraph GT9X Link wireless activity monitor (ActiGraph LLC), a triaxial accelerometer, on the nondominant wrist for 7 consecutive days. The GT9X Link has a sample rate of 30 to 100 Hz, a dynamic range of ±8G, 14-day battery life (rechargeable), 180 days/4 GB data storage, and water resistance. Data are collected at 1-minute intervals. A sensor determines whether the device is on or off the wrist. The GT9X Link provides objective 24-hour PA measures, including steps, energy expenditure, activity intensity, and participants’ posture. Accelerometry is a reliable method for assessing free-living PA (ICC=0.98) [46] and has been validated against direct observation, energy expenditure, and sedentary behavior [47,48]. ActiGraph accelerometers have been used for data collection in the National Health and Nutrition Examination surveys and are the most commonly used devices in research studies [28]. The device display screen can be disabled so that the device does not display the participant’s activity but only shows the date and time.

### Recruitment Methods

#### Identification

We have created successful, continuing relationships with older adults and staff at the planned sites and demonstrated our ability to recruit a diverse sample [49]. Staff from our community partners and clinical sites will introduce the researchers to older adults to perform the initial screening and determine study eligibility using a checklist.

#### Recruitment

Flyers will be posted at our sites, and we will participate in community activities for face-to-face recruitment. Older adults will also be recruited from their homes by local newsletters and word of mouth. The participants will complete an informed consent process before data collection. The research team will maintain a log that tracks screening, eligibility, contact, and recruitment.

### Study Procedures and Data Collection

#### Training Staff

All study personnel, including research assistants, will complete the required Collaborative Institutional Training Initiative training for the protection of human subjects. Training will be conducted by the authors (LT, JS, and JP), focusing on research with older adults in low-income settings, communication skills, setting up home visits, study protocols, completing questionnaires, performing tests, and contacting health care providers for older adults identified with a high level of depressive symptoms. We have research assistants who are bilingual and interested in research activities with Hispanic individuals. The authors (LT and JS) will train research assistants to perform balance and body composition tests. The third author (JP) will train research assistants to use accelerometer-based PA devices.

#### Data Collection

Following consent, participants will be contacted, and our team will meet with them to complete the questionnaires (eg, sociodemographic, FOF, or Patient Health Questionnaire-9) and the BBS balance performance test. Older adults will take off their shoes and stand as still as possible on the balance plate with their hands on their hips and eyes closed for 2 to 3 minutes. The participants will be stratified by the FRA matrix [12]. We will continually evaluate across the following 4 groups (rational, incongruent, irrational, and congruent) to maintain equal cell distribution (30 older adults per group):

- *Rational* FRA: low FOF (Short Fall Efficacy Scale International [FES-I] score ≤10) and aligned with normal balance (BBS score=0-30).
- *Incongruent* FRA: low FOF (Short FES-I score ≤10) despite poor balance (BBS score=31-100).
- *Irrational* FRA: high FOF (Short FES-I score >10) and normal balance (BBS score=0-30).
- *Congruent* FRA: high FOF (Short FES-I score >10) and poor balance (BBS score=31-100) [12].

In addition, participants will perform hand grip strength and dynamic balance performance tests. Hand grip strength will be measured in kilograms as the maximal isometric force achieved on a hand grip dynamometer. This test will be administered to participants sitting in a chair with their feet flat on the floor and their elbow bent at 90°. The dynamometer will be placed in the hand and adjusted so that the palm side of the grip will be at the palm and the front end will be lined up between the joints of the medial and distal phalanges. The grip size will be adjusted so that the second metacarpals are flat with a 90° bend at the knuckles. Participants will be asked to squeeze the strength gauge as hard as possible for 3 to 5 seconds. A total of 3 trials on each hand will be performed with a 30-second rest given between trials. Participants will complete the sit-to-stand test by standing up from a chair as much as possible within 30 seconds [50].

Participants will then be asked to wear the ActiGraph GT9X Link wireless activity monitor on the nondominant wrist for 7 consecutive days. Each participant will be asked to continue their normal activities while wearing the device. The participants will also be instructed and prepared for the body composition test during the second visit. They will be instructed *to avoid* exercising for 6 to 12 hours, eating for 3 to 4 hours, drinking alcohol or coffee for 24 hours, using shower or sauna, and using lotion or ointment on their hands or feet before the test. Written instructions will be provided to the participants for the use of ActiGraph and body composition tests. The questionnaires and instructions will be provided in English or Spanish by bilingual research assistants who will be trained and randomly monitored to ensure quality and consistency in administering the questionnaires and tests. The participants will be provided with a phone number of the study team if they have questions about using the activity monitoring device.

After 7 days of wearing ActiGraph, the research assistants will collect the ActiGraph GT9X Link device. Body composition testing will occur late in the morning, and all participants will be asked to empty their bladder, remove socks, shoes, and metal objects (eg, watches and jewelry) before testing. To ensure that the body is close to a resting state as possible, participants will lie down for 15 minutes before testing in the supine position. If participants select to be tested in a seated or standing position, they will be asked to sit or stand for 10 to 15 minutes before testing (based on participants’ ability and preference). Finally, participants will be asked (using our evaluation form) about their reactions to the questions and technology (ie, what they thought about the questionnaires and technology) and asked to describe any concerns or problems in wearing the device (eg, uncomfortable to wear during walking). We will provide the BBS and BIA test results, and participants will receive Can $37.43 (US $30) store gift cards upon completion of the study.

#### Participant Retention

The following strategies will be used to maximize the participants’ retention: (1) obtaining backup contact information (eg, cell phone number or email address); (2) obtaining name and contact information for a person familiar with the participant (eg, family member or neighbor); (3) making confirmation phone calls 2 days before the second visit; (4) assigning the same data collector whenever possible; and (5) making scripted check-in calls, as needed.

### Data Management and Analysis

#### Data Management and Integrity

The research assistants will enter deidentified questionnaire data into the Qualtrics Research Suite, a web-based survey tool that allows users to build and analyze web-based surveys and export data in multiple formats, including SPSS. Confidentiality will be maintained using coded data materials. Study data will be maintained on a dedicated server with password-protected access for authorized study staff and encrypted files. The research assistants will assist with data cleaning, preliminary analysis, and data preparation under the supervision of the first and fourth authors (LT and XY). We anticipate no more than 3% missing values on any one item, as data will be collected in-person on-site at 2 timepoints. The BIA device is supported by a custom software application, which connects to a virtual web-based data platform via a PC. The second author (JS) will conduct these analyses in collaboration with the first and fourth authors (LT and XY).

The ActiLife, the ActiGraph’s premier Actigraphy data analysis software platform, will be used to prepare the ActiGraph GT9X Link activity monitors for PA data collection and to download, score, and securely manage the collected data. ActiLife contains a wide selection of algorithms, including steps; energy expenditure; activity bouts such as standing, sitting, or lying down; sedentary analysis; and whether the ActiGraph device has been removed. A bout of sedentary behavior will be defined as consecutive minutes during which the ActiGraph registers less than 100 counts per minute. A break in sedentary behavior will be defined as at least 1 minute in which the counts registered are at least 100, following a sedentary bout. Activity counts and step data will be used to derive a series of PA and sedentary behavior indicator variables. We will assess the feasibility of wearing the devices by assessing the frequency of older adults who have at least 4 days of at least 10 hours of wear per day (detected using device technology), which is the standard convention [51]. The third author (JP) will conduct these analyses in collaboration with the first and fourth authors (LT and XY).

#### Data Analysis

All analyses will be performed in SPSS version 25 or SAS version 9.4, with sufficient annotation for reproducibility. Descriptive and exploratory analyses will be performed first to investigate the distributional assumptions. Although no differences are hypothesized, descriptive subgroup analyses by sex will be conducted.

##### Aim 1: Examine the Feasibility of Recruitment and Acceptability of Technologies and Procedures for Use Among Older Adults in Low-Income Settings

We will assess the ability to recruit the sample by calculating the proportions of older adults with low income (1) who are recruited out of the total screened and (2) who completed all study procedures. We will track the number of days and the time spent to recruit the sample. The results will inform the planning for future larger studies. The acceptability of the technologies and procedures will be examined based on an evaluation form (eg, what they thought about the questionnaires and technology) and their recommendations. We will identify the code, categorize participants’ responses, and determine the frequency for each category. Demographic data, including essential characteristics of older adults, will be obtained from the study participants. We anticipate no more than 3% missing values for any one item, as data will be collected in-person on-site.

##### Aim 2: Examine the Associations Among FRA, Body Composition, and PA

The hypotheses of the second aim are as follows: (1) rational FRA is associated with higher levels of PA and skeletal muscle mass and with lower levels of percentage of body fat and BMI, (2) incongruent FRA is associated with higher levels of PA and skeletal muscle mass and with lower levels of percentage of body fat and BMI, and (3) irrational and congruent FRAs are associated with lower levels of PA and skeletal muscle mass and with higher levels of percentage of body fat and BMI.

General summary statistics will be presented for continuous data, and percentages will be presented for categorical data. The participants’ demographic data will be summarized. Analyses will be performed without adjusting for any covariates, followed by analyses adjusted for age, comorbidities, depressive symptoms, and fall history. The number of comorbidities and the number of falls in the past year will be selected as covariates in the selected model because of their association with FRA, body composition, and PA. Data from InBodys10 (eg, BMI) and ActiGraph (eg, steps per day) could be modeled as continuous.

Regression models or one-way ANOVA (analysis of variance) will be used to examine the differences in continuous variables (eg, BMI and steps per day) across the 4 groups (rational, incongruent, irrational, and congruent FRAs), and categorical variables (eg, comorbidities) will be tested using chi-square tests. The rational FRA group will set as a base group, and the other 3 groups (incongruent, irrational, and congruent FRAs) will be compared with this base group. Whenever feasible, models will be adjusted for age, sex, day order, wear time (min per day), and fall history. The following variables will be added one by one to evaluate the role of mobility problems (yes or no), depression (score or category), fall risk (high or low), perceived general health (score of category), medication use (yes or no), and comorbidities (yes or no). A final model will include all significant potential variables to evaluate the associations among FRA, body composition, and PA, whenever feasible.

#### Sample Size and Power Analysis

There is no historical precedent, and there is limited information to inform the sample size for this study; therefore, the sample size estimate was based on the variable of the BBS score in our pilot study. The mean BBS scores in our pilot study were 26.44 (SD 7.91) in the high PA group and 30.62 (SD 7.78) in the low PA group (PA by self-report), and assuming a two-tail α of .05 and a power of 0.8, the estimated total sample size of 120 would allow sufficient detection of a mean difference of about 5 hypothetical units of BBS score. Furthermore, to compare the effect sizes of the different body compositions and PA measures, we performed posthoc analyses to compare the mean values between the 2 groups of PA, adjusted for the age group.

### Potential Challenges and Solutions

#### Recruitment and Retention

Although recruitment of diverse older adults to research studies is often challenging, our tailored strategies will address this issue by ensuring that our diverse research assistants are well trained and by employing a straightforward data collection process.

#### Respondent Burden

We will use questionnaires in either the English or Spanish version, based on the preferences of the older adults. Research assistants will read the questions and help older adults complete if they cannot do so.

#### Risk of Falls During Tests

We will follow the test protocol, which was used successfully in our pilot study; we had no falls when using the BBS.

### Ethical Approval and Consent to Participate

Ethical approval was obtained from the institutional review board of the University of Central Florida (protocol number 2189; September 10, 2020). All participants will be informed before their participation.

## Results

The project funding was received on September 30, 2020, and was approved by the Institutional Review Board (September 10, 2020). As of March 2021, we have enrolled 11 participants and expect the results to be published in Summer 2022.

## Discussion

Accurate FRA is essential for implementing PA programs, especially for older adults with low income. Systematic reviews found that most studies have not used objective measures of fall risk and PA [5,52]. Most studies have used self-reported measures of fall risk and PA among older adults [53-57]. Older adults who overestimate their fall risk are less likely to participate in PA [58,59]. Using AHT may eliminate recall bias associated with subjective measures [60,61]. Systematic reviews also indicate the importance of technology-based assessments for PA and to prevent falls [62,63]. This study will provide data for developing technology-based fall risk assessments to improve participation in PA, thus enhancing healthy longevity among older adults in low-income settings.

## Abbreviations

AHT: assistive health technology
BBP: BTrackS balance plate
BBS: BTrackS balance system
BIA: bioelectrical impedance analysis
FES-I: Fall Efficacy Scale International
FOF: fear of falling
FRA: fall risk appraisal
ICC: intraclass correlation coefficient
PA: physical activity
